# Diversity of Stability, Localization, Interaction and Control of Downstream Gene Activity in the Maize Aux/IAA Protein Family

**DOI:** 10.1371/journal.pone.0107346

**Published:** 2014-09-09

**Authors:** Yvonne Ludwig, Kenneth W. Berendzen, Changzheng Xu, Hans-Peter Piepho, Frank Hochholdinger

**Affiliations:** 1 Institute of Crop Science and Resource Conservation (INRES), Crop Functional Genomics, University of Bonn, Bonn, Germany; 2 Center for Plant Molecular Biology (ZMBP), Central Facilities, University of Tübingen, Tübingen, Germany; 3 Institute for Crop Science, Bioinformatics Unit, University of Hohenheim, Stuttgart, Germany; 4 College of Resources and Environment (RCBB), Southwest University, Chongqing, China; Leibniz Institute of Plant Biochemistry, Germany

## Abstract

AUXIN/INDOLE-3-ACETIC ACID (Aux/IAA) proteins are central regulators of auxin signal transduction. They control many aspects of plant development, share a conserved domain structure and are localized in the nucleus. In the present study, five maize Aux/IAA proteins (ZmIAA2, ZmIAA11, ZmIAA15, ZmIAA20 and ZmIAA33) representing the evolutionary, phylogenetic and expression diversity of this gene family were characterized. Subcellular localization studies revealed that ZmIAA2, ZmIAA11 and ZmIAA15 are confined to the nucleus while ZmIAA20 and ZmIAA33 are localized in both the nucleus and the cytoplasm. Introduction of specific point mutations in the degron sequence (VGWPPV) of domain II by substituting the first proline by serine or the second proline by leucine stabilized the Aux/IAA proteins. While protein half-life times between ∼11 min (ZmIAA2) to ∼120 min (ZmIAA15) were observed in wild-type proteins, the mutated forms of all five proteins were almost as stable as GFP control proteins. Moreover, all five maize Aux/IAA proteins repressed downstream gene expression in luciferase assays to different degrees. In addition, bimolecular fluorescence complementation (BiFC) analyses demonstrated interaction of all five Aux/IAA proteins with RUM1 (ROOTLESS WITH UNDETECTABLE MERISTEM 1, ZmIAA10) while only ZmIAA15 and ZmIAA33 interacted with the RUM1 paralog RUL1 (RUM-LIKE 1, ZmIAA29). Moreover, ZmIAA11, ZmIAA15 ZmIAA33 displayed homotypic interaction. Hence, despite their conserved domain structure, maize Aux/IAA proteins display a significant variability in their molecular characteristics which is likely associated with the wide spectrum of their developmental functions.

## Introduction

Auxin plays an eminent role in plant development and controls processes such as patterning in embryogenesis, apical dominance, gravitropism and cell elongation [1]–[3]. Three early auxin-responsive gene families have been identified and characterized including *GRETCHEN HAGEN 3* (*GH3*), *SMALL AUXIN-UP RNA* (*SAUR*) and *AUXIN/INDOLE-3-ACETIC ACID* (*Aux*/*IAA*) [4]. The *Aux*/*IAA* gene family contains 34 members in maize [5], 29 in *Arabidopsis thaliana* [6] and 31 in rice [7].

In general, Aux/IAA proteins are transcriptional repressors that share a conserved domain structure. Moreover, they are confined to the nucleus [2], [8]. Thus far, only AtIAA8 was localized in both the nucleus and the cytosol [9]. Domain I confers transcriptional repression of target genes, once the Aux/IAA and auxin response factor (ARF) protein complex interacts with promoters of downstream genes [10]. The transcriptional repressor function of Aux/IAA proteins arises from the conserved ERF-associated amphiphilic repression motif (EAR) [11], which permits the interaction with the co-repressor protein TOPLESS (TPL) and TPL-related proteins (TPR) [12]. Recent studies revealed that the former domain complex III/IV may form a type I/II Phox and Bem1p (PB1) protein-protein interaction domain [13], [14].

Protein stability of Aux/IAA proteins is conferred by the conserved degron-sequence (GWPPV) in domain II [10]. The SCF^TIR1/AFB^ complex mediates the rapid degradation of Aux/IAA proteins after binding to domain II. The importance of Aux/IAA proteins in development was demonstrated by specific point mutations [15]–[17] or deletions [18] in the degron sequence of *Aux/IAA* genes which lead to stabilized proteins [19] and defects in plant development. In *Arabidopsis thaliana*, several dominant or semi-dominant *Aux/IAA* gain-of-function mutants affected in root development were identified and analyzed [20]. The mutants *iaa28-1* [17], *iaa16-1* [*21*], *msg2-3* (*AtIAA19*) [22], *crane-1* (*AtIAA18*) [23] and *axr5-1* (*AtIAA1*) [24] display fewer lateral roots compared to the wild type. Moreover, the *slr1-1* (*AtIAA14*) mutant does not form lateral roots, and displays only few root hairs [25]. The gain-of function *Aux/IAA* mutant *bodenlos* (*bdl, AtIAA12*) lacks the embryonic root [26] while no root hairs and an agravitropic root is formed in the mutant *axr2-1* (*AtIAA7*) [27]. Finally, the semi-dominant mutant *axr3-1* (*AtIAA17*) displays reduced root elongation, impaired root gravitropism and increased adventitious rooting [28]. In rice, the mutant *mOsIAA3* displays an inhibition of seminal root formation and a decreased number of lateral- and crown roots [29]. Moreover, the gain-of-function mutant *Osiaa13* forms less lateral roots and displays a defective root gravitropic response [30].

Thus far only one *Aux/IAA* mutant is characterized in maize: the *rootless with undetectable meristem 1* (*rum1*) mutant does not initiate seminal roots and lateral roots in the primary root. The mutation results in a 26 amino acid deletion, which includes domain II. The *rum1* gene corresponds to *ZmIAA10* [18]. Subcellular localization studies revealed that the wild-type protein RUM1 is localized in the nucleus, whereas the mutated form rum1-R is detected in the nucleus and cytosol due to the partial lack of the nuclear localization signal (NLS). The RUM1 protein has a half-life time of ∼22 minutes compared to the very stable rum1-R mutant protein. It was demonstrated that RUM1 interacts with ZmARF25 and ZmARF34 [18]. In the present study, the maize Aux/IAA proteins ZmIAA2 (GRMZM2G159285), ZmIAA11 (GRMZM2G059544), ZmIAA15 (GRMZM2G128421), ZmIAA20 (GRMZM5G864847) and ZmIAA33 (GRMZM2G359924) were characterized to investigate their subcellular localization, repression of downstream gene expression, stability and interaction with other proteins and the variability and specificity of these functions.

These maize *Aux/IAA* genes were selected because they represent different properties of members of this gene family (Table 1). First, these genes have different phylogenetic ancestries and relationships. Based on their relationship to the unduplicated sorghum genome, *ZmIAA2* (subgenome 1) and *ZmIAA11* (subgenome 2) emerged before the last whole genome duplication in maize ca. 5–12 million years ago [31], while the non-syntenic genes *ZmIAA11*, *ZmIAA20* and *ZmIAA33* emerged more recently by the duplication of individual *ZmIAA* genes. Moreover, these genes encode both classes of maize Aux/IAA proteins that are separated in the maize phylogenetic tree based on their sequence of the PB1 domain (class A: ZmIAA2, ZmIAA15; class B: ZmIAA11, ZmIAA20, ZmIAA33). Furthermore, these genes display distinct root-type specific gene expression profiles and gene expression kinetics in response to auxin (Table 1). While *ZmIAA2*, *ZmIAA11*, *ZmIAA20* and *ZmIAA33* display root-type specific expression patterns, *ZmIAA15* is highly expressed in all root-types. Moreover, expression of *ZmIAA2*, *ZmIAA11* and *ZmIAA15* is significantly increased upon 1-NAA treatment within three hours (pattern A), while *ZmIAA20* and *ZmIAA33* are initially induced by auxin followed by a significant decrease of expression (pattern B, [5] and Table 1).

**Table 1:** Characteristics of maize *Aux/IAA* genes encoding proteins analyzed in this study.

Gene	maizegdb.org AC	Subgenome origin^a^	Phylogenetic class^a^	Auxin inducibility^a^	Expression in maize roots^a^
*ZmIAA2*	GRMZM2G159285_P1	1	A	A	Crown>Primary
*ZmIAA11*	GRMZM2G059544_P2	2	B	A	Lateral>All other
*ZmIAA15*	GRMZM2G128421_P1	Non-syntenic	A	A	Constitutively high
*ZmIAA20*	GRMZM5G864847_P1	Non-syntenic	B	B	Seminal>Lateral
*ZmIAA33*	GRMZM2G359924_P1	Non-syntenic	B	B	Seminal>Primary

a according to [5].

The goal of the present study was to functionally characterize five selected maize Aux/IAA proteins that displayed different attributes in earlier analyses with respect to stability, localization, protein-protein interaction and control of downstream gene activity and thus to unveil the diversity of these functions in maize.

## Material and Methods

### Plant material and growth conditions

Seeds of the maize inbred line B73 were sterilized with 6% sodium hypochlorite for 10 min and afterwards rinsed in distilled water. Subsequently, the kernels were rolled up in germination paper (Anchor paper, www.anchorpaper.com) and placed in a 10 l bucket filled with ∼2 l of distilled water. For RNA extraction, seedlings were germinated at 28 °C with a 16 h light (2700 lux) and 8 h dark cycle for six days in a plant growth chamber (Conviron Adaptis, www.conviron.com). The primary roots of these seedlings were harvested and immediately frozen in liquid nitrogen and stored at −80 °C.

For subcellular localization studies, seedlings were germinated in a plant growth chamber (Conviron Adaptis) at 28 °C with a 16 h light and 8 h dark cycle for three to four days until a 1 cm primary root was visible. Afterwards, the seedlings were transferred into an incubator (Binder, www.binder-world.com) at 26 °C in darkness for another eight to ten days. The etiolated leaves were used for protoplast isolation.

### Cloning and site-directed mutagenesis in the degron-sequence of domain II

Total RNA was extracted with the RNeasy Mini Kit (Qiagen, http://www.qiagen.com/) from 100 mg frozen material of five primary roots per biological replicate, followed by RNase-free DNAse I treatment (Fermentas, http://www.thermoscientificbio.com/fermentas/). For cDNA synthesis 1 µg of total RNA was amplified using the Revert Aid H Minus First Strand cDNA Synthesis Kit (Fermentas). To clone the open reading frames of *ZmIAA2* (GRMZM2G159285), *ZmIAA11* (GRMZM2G059544), *ZmIAA15* (GRMZM2G128421), *ZmIAA20* (GRMZM5G864847) and *ZmIAA33* (GRMZM2G359924) oligonucleotide primers were designed by PrimerPlus3 software (http://www.bioinformatics.nl/cgi-bin/primer3plus/primer3plus.cgi) and checked with NetPrimer Software (PREMIER Biosoft, http://www.premierbiosoft.com/). PCR amplicons of the *Aux/IAA* open reading frames generated by specific oligonucleotide primers were introduced into the vector pGEM-t-easy (Promega, http://www.promega.de/). Subsequently, the conserved degron-Sequence VGWPPV in domain II was mutated in all *Aux*/*IAA* genes via the GENEART site-directed mutagenesis system (Life technologies, http://www.lifetechnologies.com/). Either the first proline residue was substituted by serine (VGWSPV) or the second proline was replaced by leucine (VGWPLV). The oligonucleotides were designed according to the manufacturer's suggestions (Table S1).

### Protein stability measurement

C-terminal GFP fusion constructs were generated by amplifying either the wild-type or mutated full-length open reading frames of the *Aux/IAA* genes (*ZmIAA2*, *ZmIAA2-P264S*, *ZmIAA2-P268L*, *ZmIAA11*, *ZmIAA11-P241S*, *ZmIAA11-P245L*, *ZmIAA15*, *ZmIAA15-P226S*, *ZmIAA15-P230L*, *ZmIAA20*, *ZmIAA20-P220S*, *ZmIAA20-P224L*, *ZmIAA33*, *ZmIAA33-P232S* and *ZmIAA33-P236L*) without the stop codon using gene-specific oligonucleotides. The PCR products of the wild-type and mutated *Aux*/*IAA* gene sequences were ligated into the *Bam*HI/*Xho*I (*ZmIAA2*, *ZmIAA11* and *ZmIAA15*), *Spe*I/*Kpn*I (*ZmIAA20*) or *Xba*I/*Bam*HI (*ZmIAA33*) restriction sites of the pUC-35S-MCS-GFP vector (Lab Ac #765). All constructs of the selected *Aux/IAA* genes and a control (*35S::GFP*; Lab Ac #765) were transformed into *Arabidopsis thaliana* protoplasts as previously described [18]. The transformed protoplasts were treated with 1-NAA (1-nathphaleneacetic acid, working solution 10 µM) and cycloheximide (working solution 100 µg/ml) after 16 hours after transformation. After 1-NAA and cycloheximide treatment, samples were measured in a time course experiment (0, 10, 30, 60 and 120 min) by flow cytometry in a MoFlo cell sorter (Beckman Coulter, www.beckmancoulter.com) as previously described [18]. A 488 nm argon laser (50 mW) was used for the excitation of GFP fluorescence, the signal was recorded in FL1 (504–522 nm) and plotted against auto fluorescence in FL2 (565-605 nm). Each measurement was performed in three biological replicates. The data were documented and analyzed with Summit v4.3 software (Beckman Coulter, www.beckmancoulter.com).

### Maize protoplast isolation and transformation

Protoplast isolation of maize leaves was modified according to Sheen [32] and Nguyen et al. [33]. The middle part (6–8 cm) of etiolated 12 to 14 day-old-maize seedlings were cut in 0.5 – 1 mm strips. Subsequently, leaf digestion was performed with an enzyme solution (1.5% cellulose RS, 0.3% macerozyme R10, 0.4 M sucrose, 10 mM MES, pH 5.7) in a petri dish as described by Sheen [32]. Washing and recovering of the maize protoplasts was achieved according to Nguyen et al. [33] in 14 ml conical falcon tubes. Afterwards, quality and viability of the protoplasts was determined via the microscope Axio Lab.A1 (Zeiss, www.zeiss.de) with a Fuchs-Rosenthal bright-line objective (Labor Optik, www.lo-laboroptik.de). Protoplasts were resuspended in MMG buffer (0.4 M mannitol, 15 mM MgCl_2_ and 4 mM MES, pH 5.7) at a density of 7×10^5^ protoplasts/ml and kept on ice.

For the PEG-mediated transformation, 200 µl of isolated protoplasts, 20 µl plasmid (20–30 µg), 220 µl PEG solution (40% (v/v) PEG4000, in a 0.2 M mannitol, 0.1 M Ca(NO_3_)_2_ buffer) were combined in a 14 ml conical tube and thoroughly mixed. The mixture was incubated at RT for 15 min, and later diluted to 10 ml with wash buffer (5 mM NaCl, 5 mM glucose, 125 mM CaCl_2_⋅2H_2_O, 154 mM KCl). After centrifugation at 200 g for 1 min, the protoplast pellet was dissolved in 200 µl WI solution (0.6 M mannitol, 4 mM MES pH 5.7, and 4 mM KCl) and incubated in the dark at 26 °C ON.

### Subcellular localization

The C-terminal GFP fusion constructs established for the protein stability determination were also used for subcellular localization experiments in isolated maize leaf protoplasts. For *ZmIAA11*, *ZmIAA11-P241S*, *ZmIAA11-P245L*, *ZmIAA20*, *ZmIAA20-P220S* and *ZmIAA20-P224L*, additional N-terminal GFP fusion constructs were generated. To construct the pUC-35S-GFP-MCS the open reading frame of the GFP without stop codon was amplified with the oligonucleotides GFP*-Xba*I-F and GFP-*Sac*I-R2 using the pUC-35S-MCS-GFP vector as template (Lab Ac #765). The GFP PCR-products were inserted in the *Xba*I/*Sac*I site of the backbone vector. The MCS was generated by PCR amplification (oligonucleotide primers: MCS4-*Sac*I-F and MCS4-*Sac*I-R) with the pUC-SYPNE vector (Lab Ac #519) as template. The MCS was inserted at the *Sac*I site of the recipient vector (Lab Ac #949). The vector sequences were confirmed by sequencing.

To construct the N-terminal GFP fusions, the amplified PCR-products were inserted into the *Bam*HI/*Xho*I (oligonucleotide primers: ZmIAA11-*Bam*HI-F2 and ZmIAA11-*Xho*I-R) and *Spe*I/*Xho*I (oligonucleotide primers: ZmIAA20-SpeI-F and ZmIAA20-XhoI-R) restriction sites of the pUC-35S-GFP-MCS vector (Lab Ac #979). These constructs and the control (35S::GFP-MCS or 35S::MCS-GFP) were transformed into maize protoplasts and documented by confocal laser scanning microscopy (Zeiss LSM-780, attached to an Axio Observer Z1, Carl Zeiss Microscopy, www.zeiss.com). 4',6-diamidino-2-phenylindole (DAPI) was used as nuclear counterstain.

### Transient luciferase expression assay

The effector vector (pUC-35S-TMV-GAL4BD-MCS-NosT, Lab Ac #988) was generated by amplifying the TMV-GAL4-DBD PCR-fragment (oligonucleotide primers: TMV-*Xba*I-F and GAL4-*Sac*I-R) from the donor vector (pUC-35S-GAL4-DBD-cYFP, Lab Ac # 936). The PCR-product was inserted into the restriction site *Xba*I/*Sac*I of the backbone vector (Lab Ac #765), which provided the 35S promoter and the Nos terminator to construct an intermediate vector (pUC-35S-TMV-GAL4BD-Nos-T, Lab Ac #954). Moreover, a MCS was obtained from the pUC-SYPNE vector (Lab Ac # 519) by PCR with the oligonucleotides MCS4-*Sac*I-F and MCS4-*Sac*I-R. The intermediate vector (Lab Ac #954) was digested with *Sac*I and the MCS-product was inserted. The effector vector sequence was confirmed by sequencing. PCR fragments of the wild-type *Aux/IAA* genes were amplified and ligated into the *Bam*HI/*Kpn*I (*ZmIAA2*, *ZmIAA11* and *ZmIAA33*), *Bam*HI/*Xma*I (*ZmIAA15*) and *Spe*I/*Kpn*I (*ZmIAA20*) restriction sites of pUC-35S-TMV-GAL4BD-MCS-NosT (effector plasmid, Lab Ac #988).

The reference plasmid encoding for *renilla* LUC (Luciferase) and the reporter plasmid encoding for *firefly* LUC, both driven by a 35S Cauliflower mosaic virus (CaMV) promoter, and the effector plasmid were co-transformed into *Arabidopsis* protoplast. For the transformation 1 µg plasmid DNA of the reference plasmid, 3 µg of reporter and 5 µg of effector plasmid were used, which were determined by a dose-response assay as described in the Dual-Luciferase reporter assay system manual (Promega, www.promega.de).

To determine the luciferase activities, the Dual-Luciferase reporter assay system (Promega) was used and quantified in a plate reader (CLARIOstar, www.bmglabtech.com). Three biological replicates were measured per *Aux/IAA* gene and luciferase activity of each transformant was determined three times independently. The values were normalized with the corresponding values of the internal control *renilla* LUC, which minimized the experimental variability introduced by the variation of the transfection efficiency and the protoplast viability. Differences of the relative luciferase activities were determined by a one-sided Student's t-test.

### Bimolecular fluorescence complementation and flow cytometry

Interaction studies were conducted by bimolecular fluorescence complementation (BiFC) as described by Walter et al. [34]. Fusion plasmids of *ZmIAA2*, *ZmIAA11*, *ZmIAA15*, *ZmIAA20* and *ZmIAA33* were generated with the C or N-terminal fragment of YFP. Donor plasmids encoding the open reading frame without stop codon of the genes of interest (*ZmIAA2*: Lab Ac #880, *ZmIAA11*: Lab Ac #883, *ZmIAA15*: Lab Ac #887, *ZmIAA20*: Lab Ac #913, *ZmIAA33*: Lab Ac #891) were digested with *Bam*HI/*Xba*I (*ZmIAA33*), *Bam*HI/*Xho*I (*ZmIAA2*, *ZmIAA11*, *ZmIAA15*) or *Spe*I/*Kpn*I (*ZmIAA20*) to release the *Aux/IAA* open reading frames. These open reading frames were introduced into the corresponding restriction sites of the recipient vectors pUC-SPYNE-152 ([34] and Lab Ac #518) and pUC-SPYCE ([35] and Lab Ac #519). Likewise, interactions were studied with RUM1 (ZmIAA10: GRMZM2G037368) and RUL1 (ZmIAA29: GRMZM2G163848). The split-YFP constructs of RUM1 (Lab Ac #528 and 531) were generated as described in von Behrens et al. [18]. The full-length open reading frame of *rul1* was amplified with the oligonucleotide primer *rul1*-*BamH*I-fw and *rum1*-*Kpn*I-rv. The donor vectors pUC-SYPCE and pUC-SYPNE-152 were digested and the PCR product ligated into the restriction site *Bam*HI/*Kpn*I. The sequences of the C- or N-terminal fragment YFP –RUL1 constructs (Lab Ac #529 and 530) were validated via sequencing. As a negative control an uncharacterized protein of the *barwin* gene family (*BARW*, GRMZM2G117942) was used. The corresponding C and N-terminal YFP fusion constructs were co-transformed into *Arabidopsis* protoplasts, according to Berendzen et al. [36] and analyzed by flow cytometry. In total 76 different samples were measured in three replicates. Per 96 well plate one biological replicate of each sample was measured.

A linear model was fitted with an additive effect for treatment, corresponding to the protein interaction partners. In order to normalize the data between different plates, an additive effect for plates was fitted. Inspection of the studentized residuals suggested that a logarithmic transformation stabilized the variance and led to an approximate normal distribution. Thus, all analyses were performed on the logarithmic scale. Note that an additive model on the transformed scale corresponds to a multiplicative model on the original scale. According to this model, responses were expected on the original scale of the different treatments on one plate to be a multiple of the responses on another plate. This expectation also met with the observation.

Each interacting protein pair was compared to the mean of its two controls by specifying the corresponding linear contrast. In this contrast, the protein interacting pair was assigned a coefficient of 2, whereas the two controls were each assigned coefficients of -1. T-tests were performed for all contrasts of interest. The *p*-values were adjusted for multiplicity using the simulation method of Edwards and Berry [37]. The family-wise type I error rate was set at α = 5%.

To confirm the interaction results obtained from the quantitative BiFC assays, split-YFP experiments were performed in maize protoplasts. To confirm the expression of the proteins, Western blot experiments were performed as previously described [18].

## Results

### Stability of maize Aux/IAA proteins is very variable

**Figure 1:**
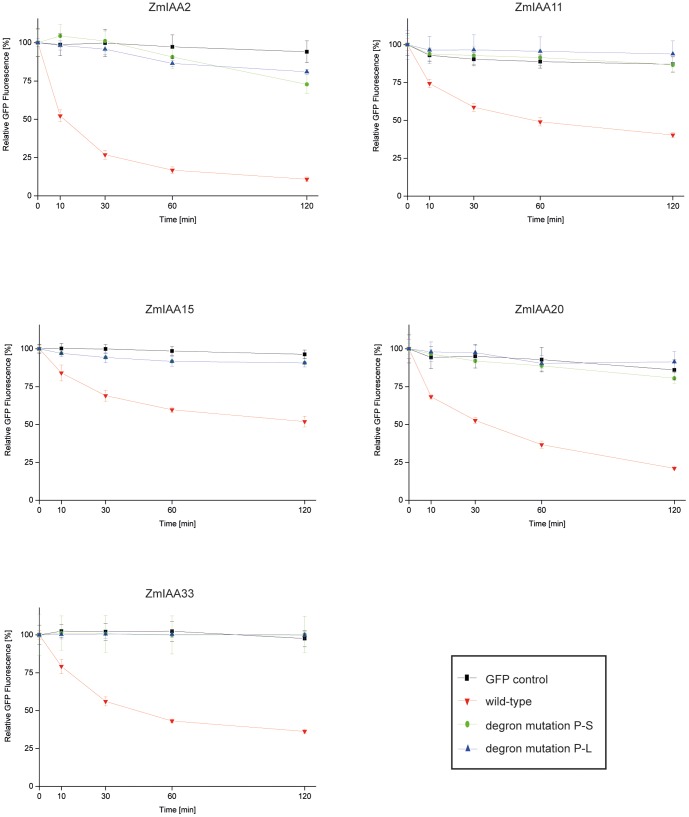
Degradation assay of maize Aux/IAA-GFP wild-type fusion proteins and Aux/IAA-GFP proteins containing mutations in their degron sequence. Detection of relative GFP-fluorescence of the GFP control, wild-type and two mutated forms of Aux/IAA proteins after 1-NAA and cycloheximide treatment prior to recording a two hour time course in *Arabidopsis* protoplasts. Red: wild-type, green: substitution of first proline by serine, blue: substitution of second proline by leucine, black: GFP control.

The rapid degradation of Aux/IAA proteins in response to high auxin levels is crucial for auxin signal transduction and thus controls the activity of downstream gene activity. To compare the stability kinetics of the five selected maize Aux/IAA proteins, C-terminal green fluorescent protein (GFP) fusions were generated. Two different point mutations were introduced into the degron sequence (VGWPPV) of the Aux/IAA proteins. These mutations resulted in a substitution of the first proline (P) by serine (S), or the exchange of the second proline (P) by leucine (L). In *Arabidopsis*, it was demonstrated that these specific substitutions resulted in more stable proteins compared to the wild-type [19], [38]. The relative GFP fluorescence intensity was determined over a time period of two hours in *Arabidopsis* protoplasts by flow cytometry, after treatment with the synthetic auxin 1-NAA and cycloheximide. As a control, the GFP fluorescence intensity of free GFP was measured. For each of the five Aux/IAA proteins, different average half-life times were determined: ZmIAA2-GFP had an average half-life time of ∼11 minutes, ZmIAA20-GFP and ZmIAA33-GFP displayed a half-life time of ∼40 minutes and ZmIAA11-GFP and ZmIAA15-GFP displayed the longest average half-life times of ∼60 and ∼120 minutes, respectively (Figure 1). As a consequence of the substitution of the amino acid P to S or L in the degron sequence, all modified Aux/IAA-GFP proteins were significantly more stable than their respective wild-type protein during the whole time course of 120 minutes. Most mutated Aux/IAA-GFP proteins appeared slightly less stable than the GFP control, as indicated by a minimal decrease of the measured GFP fluorescence intensity over time. However, these values were not significantly different from the GFP control. The degradation of the Aux/IAA-GFP proteins was independently confirmed by Western blot experiments (Figure S2).

### Subcellular localization of Aux/IAA proteins

**Figure 2:**
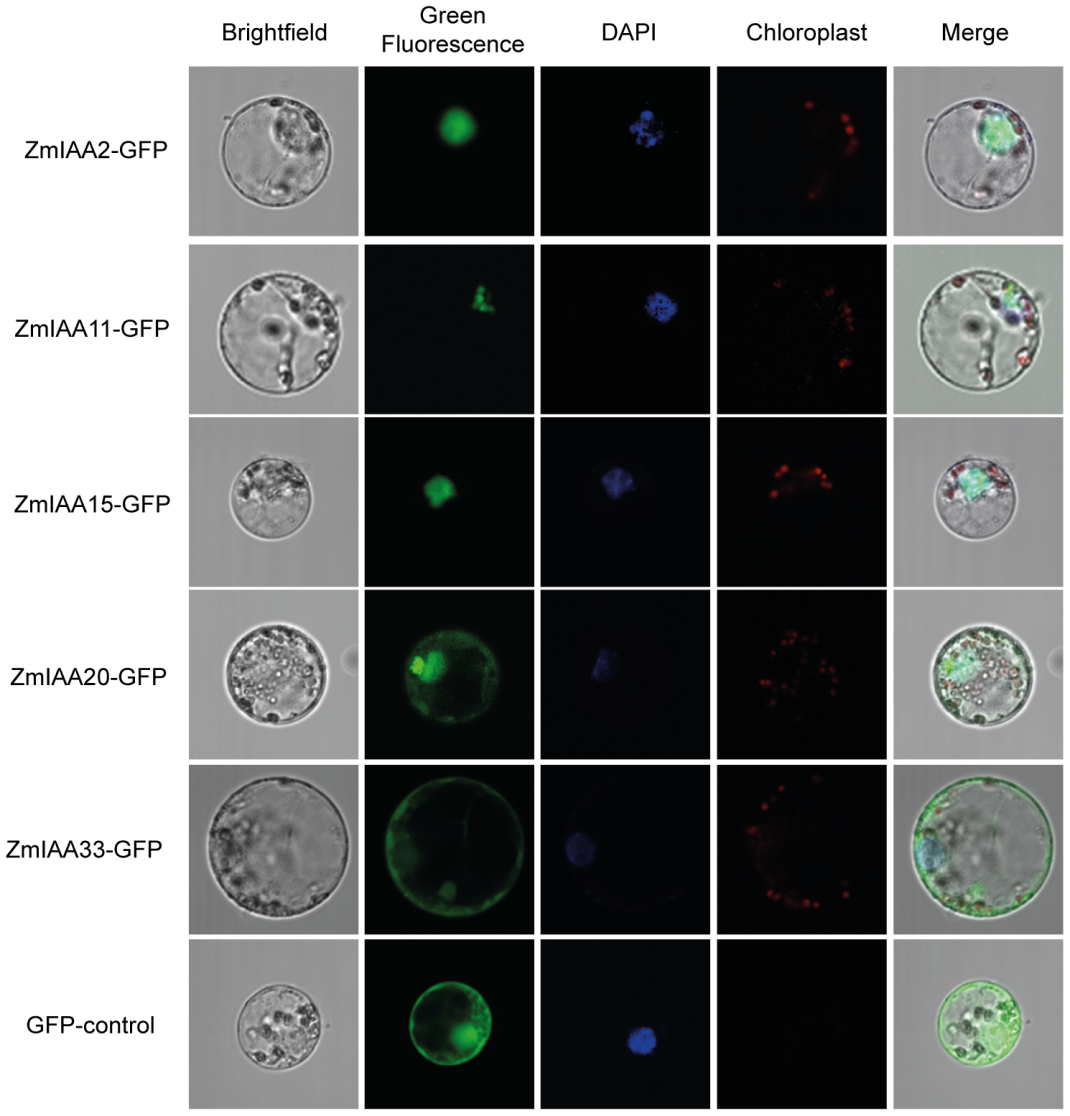
Subcellular localization of Aux/IAA-GFP fusion proteins in maize protoplasts by GFP-fluorescence. ZmIAA2, ZmIAA11 and ZmIAA15 were confined to the nucleus. Cytosolic and nuclear localization was identified for ZmIAA20 and ZmIAA33. Subcellular localization of mutated versions of the five Aux/IAA proteins and N-terminal GFP fusions that confirmed the subcellular localization of the C-terminal fusions are displayed in Figure S3.

Aux/IAA proteins are transcriptional repressors and therefore act in the nucleus. To survey the subcellular localization of the selected Aux/IAA proteins, C-terminal GFP-fusion proteins of wild-type and the two mutated forms were generated for all five *Aux/IAA* genes and transiently expressed in maize mesophyll protoplasts. The wild-type GFP-fusion proteins of ZmIAA2-GFP, ZmIAA11-GFP and ZmIAA15-GFP were exclusively accumulated in the nucleus. In contrast, ZmIAA20-GFP and ZmIAA33-GFP were localized in both the nucleus and the cytosol in maize protoplasts (Figure 2). Both, ZmIAA20 and ZmIAA33 have an incomplete NLS lacking the amino acids KR in the first part of the bipartite NLS (Figure S1) which might explain their subcellular localization pattern. ZmIAA11-GFP and ZmIAA20-GFP displayed a compartmentalized expression in the nucleus (Figure 2). To investigate if the compartmentalization in the nucleus is a consequence of the C-terminal GFP fusion, N-terminal GFP-fusion proteins of wild-type and their mutated forms were generated. Identical results, i.e. compartmentalized GFP signals, were observed for GFP-ZmIAA11 and GFP-ZmIAA20. Moreover, the N-terminal GFP-fusion GFP-ZmIAA20 displayed the same localization in both nucleus and cytoplasm as the C-terminal GFP-fusion ZmIAA20-GFP. All mutated proteins displayed the same subcellular localization as the five wild-type proteins (Figure S3).

### All five Aux/IAA genes are active repressors of downstream gene expression

Transcriptional repression of Aux/IAA proteins is mediated by domain I. To investigate the regulation of downstream genes by the five maize Aux/IAA proteins, the Dual-Luciferase reporter assay was applied in *Arabidopsis* protoplasts. This system allows to test if proteins containing a GAL4-DBD (DNA Binding Domain), function as repressors or activators of downstream gene expression by their control of *firefly* luciferase activity by binding to a GAL4-UAS (Upstream Activating Sequence) of a reporter gene. *Renilla* luciferase activity is used as an internal reference to normalize the determined values.

**Figure 3:**
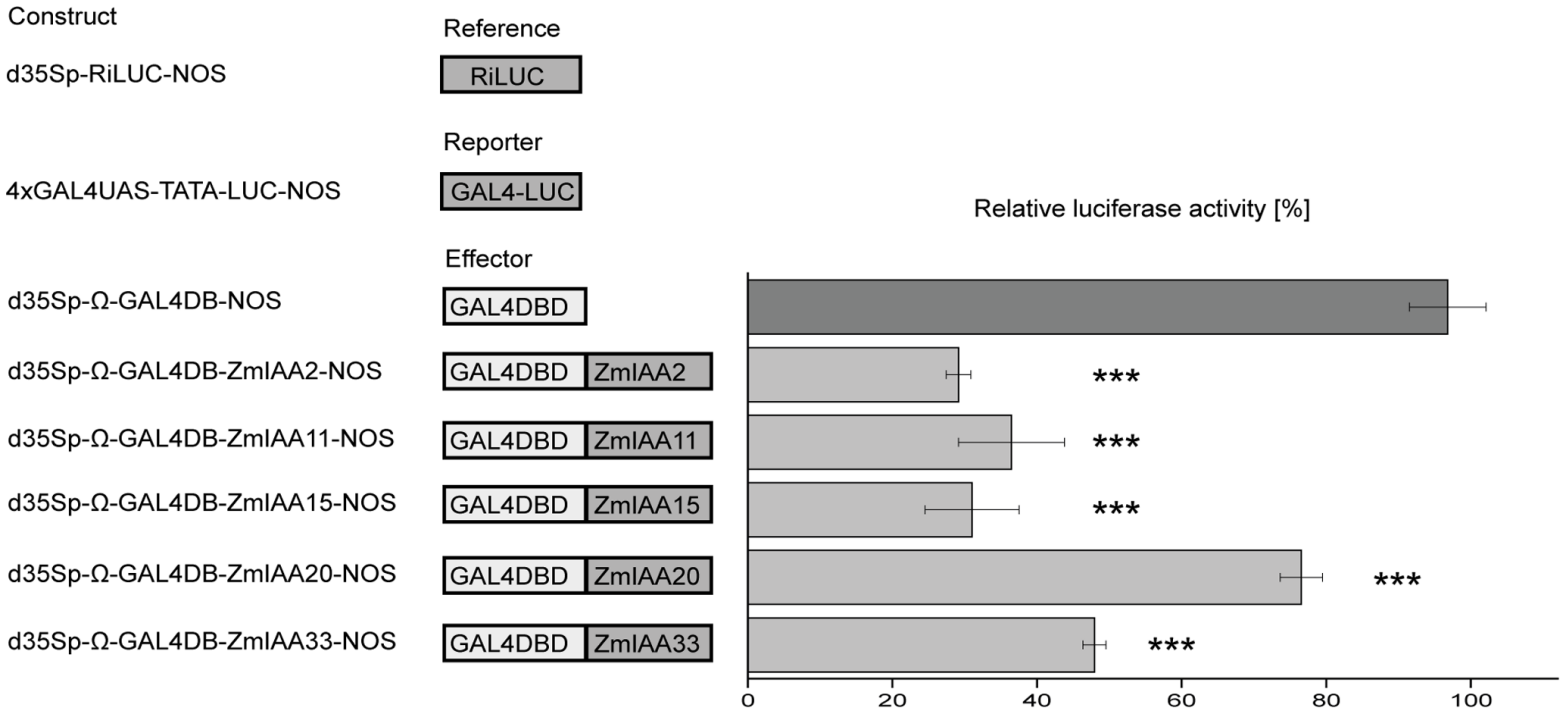
Repression of a *firefly* luciferase reporter gene by Aux/IAA proteins fused to a GAL4 DNA binding domain. Aux/IAA proteins fused to GAL4 DNA binding domains were co-transformed with a *firefly* luciferase reporter into *Arabidopsis* protoplasts. All luciferase activities were normalized relative to the empty effector vector (GAL4-DBD) and an internal luciferase control (*renilla* luciferase). Error bars: SD, n = 3, *p*≤0.001.

Fusion proteins containing a N-terminal yeast GAL4 DNA-binding Domain and full-length wild-type Aux/IAA proteins at the C-terminus were used as effectors (Figure 3). The reporter plasmid encoded *firefly* luciferase (LUC) proteins driven by a minimal TATA box of the CaMV 35S promoter combined with an upstream GAL4-UAS binding site. *Arabidopsis* protoplasts were co-transformed with three plasmids including the reference plasmid encoding for *renilla* LUC, a reporter plasmid encoding for *firefly* LUC and either the control GAL4-DBD effector plasmid or effector plasmids encoding for one of the five GAL4-DBD-ZmIAA proteins. Relative *firefly* luciferase activity of the Aux/IAA proteins was measured and quantified relative to the control GAL4-DBD protein. All GAL4-DBD-ZmIAA proteins exerted a significant reduction of relative luciferase activity on the downstream reporter gene compared to the GAL4-DBD control (Figure 3). GAL4-DBD-ZmIAA2 and GAL4-DBD-ZmIAA15 displayed the strongest reduction, with only ∼30% residual relative luciferase activities. Moreover, the relative luciferase activity was ∼36% for GAL4-DBD-ZmIAA11 and ∼48% for GAL4-DBD-ZmIAA33, whereas GAL4-DBD-ZmIAA20 displayed ∼77% activity compared to GAL4-DBD control (Figure 3). These results suggest a significant transcriptional repression of downstream genes by the tested Aux/IAA proteins but also a considerable variability between the repressor function of different Aux/IAA proteins.

### Homo and heterointeraction of maize Aux/IAA proteins including RUM1 and its paralog RUL1

**Figure 4:**
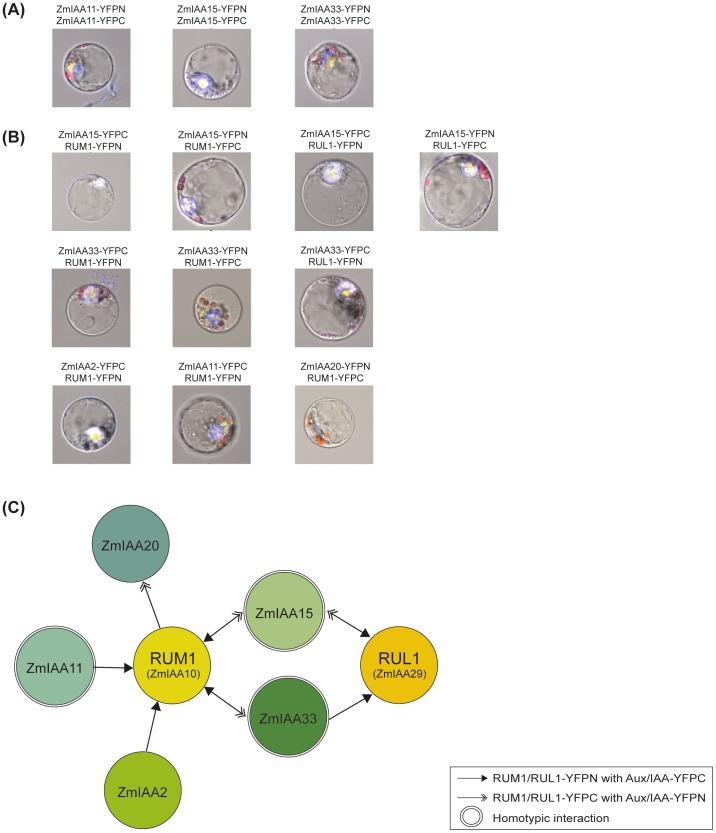
Homo- and heterointeraction of Aux/IAA proteins in BiFC experiments visualized by confocal microscopy. **(A)** Homotypic protein interactions of Aux/IAA proteins were identified for ZmIAA11, ZmIAA15 and ZmIAA33 but not for ZmIAA20 and ZmIAA2. **(B)** Heterotypic interactions of RUM1 were detected for all five tested Aux/IAA proteins. RUL1 specifically interacted with ZmIAA15 and ZmIAA33. **(C)** Summary of the quantitative BiFC data obtained by flow cytometry (see Table 2) and the verification of these results by qualitative BiFC experiments surveying protoplasts under a confocal microscope (Figure 4A and B). The arrowhead types are indicating the interacting partners. A full arrowhead describes an interaction of RUM1/RUL1-YFPN with Aux/IAA-YFPC, while a double arrowhead denotes an interaction of RUM1/RUL1-YFPC with Aux/IAA-YFPN. Homotypic interactions of ZmIAA11, ZmIAA15 and ZmIAA33 are highlighted with a double circle.

The PB1 domain complex of Aux/IAA proteins allows interaction with other Aux/IAA proteins or auxin response factors (ARFs). To examine homotypic interactions of the five Aux/IAA proteins surveyed in the present study, quantitative bimolecular fluorescence complementation (BiFC) experiments were performed by flow cytometric analysis of transformed protoplasts. Subsequently, the results obtained in flow cytometric analyses were independently confirmed by analyzing BiFC interactions in individual maize protoplasts under a Zeiss LSM 780 (attached with Axio Observer Z1, Zeiss) confocal laser scanning microscope. Homotypic interaction of the Aux/IAA proteins ZmIAA11, ZmIAA15 and ZmIAA33 was detected in quantitative FACS experiments (Table 2) and confirmed in subsequent confocal laser scanning microscopic studies of individual protoplasts (Figure 4A). Homotypic interactions of ZmIAA2 and ZmIAA20 were neither detected by flow cytometry nor when analyzing BiFC interactions in individual maize protoplasts in a confocal laser scanning microscope (Table 2). Moreover, interaction of the five maize Aux/IAA proteins studied here was tested with the previously characterized paralogous RUM1 (ZmIAA10) and RUL1 (ZmIAA29) proteins involved in maize lateral and seminal root formation. Interactions were detected for all five Aux/IAA proteins with RUM1, whereas only ZmIAA15 and ZmIAA33 interacted with RUL1 (Figure 4B and Table 2). These interactions were validated quantitatively by flow cytometry and confirmed in individual protoplasts by confocal imaging. None of the negative controls using the BARW protein, which does not interact with Aux/IAA proteins, revealed an interaction with any of the five Aux/IAA proteins (Figure S4). Expression of the proteins in the interaction studies was confirmed by Western blot analyses (Figure S5). A summary of all observed Aux/IAA interactions is provided in Figure 4C).

**Table 2:** Statistical evaluation of BiFC data using a lineal model for logarithmized data and simulation-based multiple comparisons by Edwards and Berry [37].

Interacting proteins	Value (log scale)^a^	*p*-value^b^	Adj *p-*value^c^	significance^d^
ZmIAA2-YFPN/ZmIAA2-YFPC	0.80	0.41	1	ns
ZmIAA2-YFPN/RUM1-YFPC	2.30	0.02	0.60	ns
RUM1-YFPN/ZmIAA2-YFPC	4.06	<0.01	<0.01	*
ZmIAA2-YFPN/RUL1-YFPC	1.43	0.14	1.00	ns
RUL1-YFPN/ZmIAA2-YFPC	2.86	<0.01	0.17	ns
ZmIAA11-YFPN/ZmIAA11-YFPC	10.06	<0.01	<0.01	*
ZmIAA11-YFPN/RUM1-YFPC	0.69	0.47	1	ns
RUM1-YFPN/ZmIAA11-YFPC	5.22	<0.01	<0.01	*
ZmIAA11-YFPN/RUL1-YFPC	0.87	0.37	1	ns
RUL1-YFPN/ZmIAA11-YFPC	3.16	<0.01	0.07	ns
ZmIAA15-YFPN/ZmIAA15-YFPC	11.42	<0.01	<0.01	*
ZmIAA15-YFPN/RUM1-YFPC	7.05	<0.01	<0.01	*
RUM1-YFPN/ZmIAA15-YFPC	7.93	<0.01	<0.01	*
ZmIAA15-YFPN/RUL1-YFPC	5.29	<0.01	<0.01	*
RUL1-YFPN/ZmIAA15-YFPC	6.74	<0.01	<0.01	*
ZmIAA20-YFPN/ZmIAA20-YFPC	1.65	0.09	0.99	ns
ZmIAA20-YFPN/RUM1-YFPC	3.71	<0.01	<0.01	*
RUM1-YFPN/ZmIAA20-YFPC	−0.94	0.33	1	ns
ZmIAA20-YFPN/RUL1-YFPC	2.14	0.03	0.75	ns
RUL1-YFPN/ZmIAA20-YFPC	−0.53	0.58	1	ns
ZmIAA33-YFPN/ZmIAA33-YFPC	9.64	<0.01	<0.01	*
ZmIAA33-YFPN/RUM1-YFPC	5.09	<0.01	<0.01	*
RUM1-YFPN/ZmIAA33-YFPC	7.57	<0.01	<0.01	*
ZmIAA33-YFPN/RUL1-YFPC	2.65	0.01	0.30	ns
RUL1-YFPN/ZmIAA33-YFPC	6.80	<0.01	<0.01	*

a value after using lineal model for logarithmized data.

b t-test of value (log scale).

c *p*-value adjusted for simulation-based multiple comparisons by Edwards and Berry [37].

d ns: not significant; *: significant (adjusted p-value <0.01).

## Discussion

The maize genome contains 34 *Aux*/*IAA* genes [5], which encode plant-specific transcriptional regulators of auxin signaling [39]. Canonical Aux/IAA proteins consist of domains I, II and PB1 and contain a bipartite NLS [40]. The five Aux/IAA proteins characterized in the present study (ZmIAA2, ZmIAA11, ZmIAA15, ZmIAA20, and ZmIAA33) display the canonical domain structure.

Domain II of Aux/IAA proteins, contains a degron sequence, which confers the rapid degradation of Aux/IAA proteins by interacting with the SCF^TIR1^ complex [41]. As a consequence, Aux/IAA proteins are short-lived proteins with a half-life time of ∼6 to 8 min in pea [42] and ∼10 to ∼80 min in *Arabidopsis thaliana* [38], [43]. Specific point mutations in the short degron sequence VGWPPV stabilizes these Aux/IAA proteins [19], [28], [38], [44]. The half-life time of maize RUM1 (ZmIAA10) has been previously determined as ∼22 min [18]. In the present study, half-life times of maize Aux/IAA proteins ranged from ∼11 min (ZmIAA2), to ∼120 min (ZmIAA15) in Arabidopsis protoplasts. ZmIAA20 and ZmIAA33 displayed a half-life time of ∼40 min. Both genes are closely related to each other and display 77% identity in their amino acid sequence. Similarly, *Arabidopsis* Aux/IAA proteins, that share a high similarity outside the conserved domain II, displayed similar half-life times [44], suggesting that the degron sequence is required for the degradation of Aux/IAA proteins but that the half-life time of these proteins is also determined by sequences outside the degron motif [44]. The half-life time of ZmIAA15 (∼120 min) exceeded the most stable *Arabidopsis* Aux/IAA proteins AXR3/IAA17 [28] and IAA28 at ∼80 min [44]. The difference in stability of the different maize Aux/IAA proteins tested here might be a result of tissue- or organ-specific regulation by yet unidentified proteins or unidentified stabilons of degrons outside of domain II [44]. However, these significant differences in maize Aux/IAA protein half-life times, should rather be considered as relative than absolute because the data was obtained in heterologous Arabidopsis protoplasts. In line with the predicted molecular function of the degron motif, point mutations, substituting the first P by S or the second P by L, in the sequences of the five Aux/IAA proteins analyzed here resulted in proteins that displayed almost no degradation (<10%) over a time period of 120 min. Similar results were described for the mutated maize ZmIAA10 protein rum1-R in which the degron sequence was completely deleted [18].

Commonly, Aux/IAA proteins hold a bipartite nuclear localization signal (NLS), which directs them into the nucleus. Nuclear localization was observed for the Aux/IAA proteins ZmIAA2, ZmIAA11 and ZmIAA15 and their mutated forms. Previously we demonstrated that the RUM1 protein (ZmIAA10) is also localized in the nucleus [18]. Similar results were described for the Aux/IAA proteins AXR2-GUS and AXR3NT-GUS in *Arabidopsis*, which were localized in the nucleus [28], [38]. Moreover, nuclear localization was observed for the *Arabidopsis* Aux/IAA proteins IAA26 [45], AXR2, MSG2, SLR [46], BDL [47] and IAA17 [28], [48]. In contrast, ZmIAA20 and ZmIAA33 were not exclusively expressed in the nucleus but also in the cytosol. This might be a result of the two missing conserved amino acids KR of the first part of the NLS. In *Arabidopsis*, only one Aux/IAA protein, AtIAA8, was thus far localized in the cytosol and nucleus [9]. A function of Aux/IAA proteins in the cytosol has been described recently by the interaction of LSD1 (LESIONS SIMULATING DISEASE RESISTANCE 1) with AtIAA8 [49]. LSD1 is a cytosolic protein which negatively controls cell death and disease resistance. Furthermore, it sequesters the transcriptional factor AtbZIP10 [50]. Hence, ZmIAA20 and ZmIAA33 might not exclusively function in the transcriptional regulation of auxin signaling but may also play a role in cytosolic processes.

All Aux/IAA proteins analyzed in the present study repressed downstream gene expression as demonstrated by a dual luciferase-assay. Relative luciferase activity was repressed between ∼23% (GAL4-DBD-ZmIAA20) and ∼70% (GAL4-DBD-ZmIAA2 and GAL4-DBD-ZmIAA15) by the Aux/IAA effectors in comparison to the GAL4-DBD control. Previously we demonstrated that maize RUM1 (ZmIAA10) exerted a transcriptional repression of ∼59% on downstream target genes [18]. These values should rather be considered relative than absolute because these experiments have been performed in heterologous Arabidopsis protoplasts. In *Arabidopsis*, the repressor function of domain I of sixteen Aux/IAA proteins was determined by GUS reporter assays. These Aux/IAA proteins repressed the GUS reporter gene between 51% to 89% relative to the control [51]. In *Arabidopsis*, a correlation was observed between Aux/IAA protein stability in wild-type versus mutant proteins and their repression of early auxin response genes. Stabilizing mutations in domain II, resulted in enhanced repression of downstream targets by these proteins. In contrast, mutations in domain I and III were able to partially reverse repressor function [51].

Protein-protein interaction studies revealed homo- and heterotypic interaction of maize Aux/IAA proteins. Homotypic interaction of ZmIAA11, ZmIAA15 and ZmIAA33 was determined by BiFC experiments in Arabidopsis protoplasts which were quantified by flow cytometry and subsequent independent verification by confocal microscopy in maize protoplasts (Figure 4A and Table 2). In yeast-two hybrid experiments, homotypic interactions of *Arabidopsis* IAA1 and IAA2 and IAA4 of pea were described [52]. Protein-protein interaction of five Aux/IAA proteins with RUM1 (ZmIAA10) and RUL1 (ZmIAA29) were quantified in BiFC experiments by flow cytometry in Arabidopsis protoplasts. Independent confirmation was performed by confocal microscopy in maize protoplasts (Figure 4B and Table 2). Hetero-interactions were observed for all Aux/IAA proteins tested with RUM1, while only two of five ZmIAA proteins (ZmIAA15 and ZmIAA33) interacted with RUL1. The *Aux*/*IAA* gene *rul1* is expressed ∼84 fold higher than *rum1*. Likewise *ZmIAA15* is one of the highest expressed maize *Aux*/*IAA* gene in contrast to *ZmIAA2*, *ZmIAA20* and *ZmIAA33* which display relatively low expression levels [5]. However, the expression levels of the different *Aux*/*IAA* genes were not correlated with the interaction between them suggesting specificity of Aux/IAA interactions irrespective of their expression levels. In yeast-two-hybrid experiments, *Arabidopsis* IAA1 interacted with 15 different Aux/IAA proteins [52]. It was suggested that several hundred interactions might be responsible for the diversity of physiological and morphological responses of these transcription factors based on the observation of differential spatial and temporal expression patterns of *Aux/IAA* genes in plants [52]. The functional characterization of the Aux/IAA proteins revealed a wide variability and specificity of different molecular functions of Aux/IAA proteins. In combination with specific expression patterns in root and shoot tissues, these results support the notion of a wide variety of Aux/IAA protein functions in plant growth and development.

## Supporting Information

Figure S1 Alignment of the maize Aux/IAA protein sequences relevant for this study, including RUM1 (ZmIAA10) and RUL1 (ZmIAA29). The protein sequences were compared by multiple alignments with ClustalOmega. The canonical domains are highlighted by grey shading. The bipartite nuclear localization signal is encircled in red.
(PDF)

Figure S2 Confirmation of Aux/IAA degradation by Western blot experiments. Western blot analysis of Aux/IAA-GFP constructs after 1-NAA (10 µM) and cycloheximide (100 µg/ml) treatment using an anti-HA antibody. Protein abundance was quantified at three different time points (0, 30 and 120 min). Lane order for ZmIAA15 was manually rearranged to correct for the initial omission of the 30 min sample which was loaded on the same gel in a lane to the right.
(PDF)

Figure S3 Subcellular localization studies of maize Aux/IAA proteins and their mutated forms in maize protoplasts. Detailed summary of the subcellular localization studies of maize Aux/IAA wild-type proteins and two mutated protein forms of each protein in maize protoplasts. As a control, the empty GFP construct constitutively expressing GFP, was localized in the cytosol and nucleus. ZmIAA2, ZmIAA11 and ZmIAA15 were confined to the nucleus. The specific point-mutations in the degron-sequence did not affect localization. For ZmIAA20 and ZmIAA33, wild-type and mutated proteins were localized in the cytosol and the nucleus. To investigate if the compartmentalization of the GFP signal is a result of C-terminal GFP, N-terminal GFP fusion constructs of ZmIAA11 and ZmIAA20 with their mutated forms were tested. The localization study displayed an accumulation in a compartmental manner in the nucleus as described before.
(PDF)

Figure S4 Negative controls for protein-protein interaction studies in maize protoplasts. In maize protoplasts split-YFP experiments were conducted to demonstrate that Aux/IAA proteins do not interact with the control protein BARW. Red: auto fluorescence, blue: DAPI counterstaining.
(PDF)

Figure S5 Confirmation of fusion protein expression in protein-protein interaction studies. Expression of the fusion proteins was demonstrated by Western blot experiments. The positions of the expressed proteins are indicated with red stars to the right of the corresponding lane. Anti-Myc antibodes were used for the detection of YFPN-152 and anti-HA for the detection of YFPC.
(PDF)

Table S1 Oligonucletodide primer used in this study. The enzyme restriction sites are indicated by small letters.
(XLSX)

## References

[1] Went FW, Thimann KV (1937) Phytohormones. Macmillan, New York, USA.

[2] Abel S, Nguyen MD, Theologis A (1995) The *PS-IAA4/5-like* Family of Early Auxin-inducible mRNAs in *Arabidopsis thaliana* . J Mol Biol 251: 533–549. 7658471 10.1006/jmbi.1995.0454

[3] Abel S, Theologis A (1996) Early genes and auxin action. Plant Physiol 111: 9–17. 8685277 10.1104/pp.111.1.9 PMC157808

[4] Guilfoyle T (1999) Auxin-regulated genes and promoters. In: Hooykaas PJJ, Hall MA, Libbenga KR, (eds), Biochemistry and molecular biology of plant hormones Elservier, Amsterdam, The Netherlands.

[5] Ludwig Y, Zhang Y, Hochholdinger F (2013) The Maize (*Zea mays* L.) *AUXIN/INDOLE-3-ACETIC ACID* Gene Family: Phylogeny, Synteny, and Unique Root-Type and Tissue-Specific Expression Patterns during Development. PLoS ONE 8: e78859. 24223858 10.1371/journal.pone.0078859 PMC3815225

[6] Liscum E, Reed JW (2002) Genetics of Aux/IAA and ARF action in plant growth and development. Plant Mol Biol 49: 387–400. 12036262

[7] Jain M, Kaur N, Garg R, Thakur J, Tyagi A, et al (2006) Structure and expression analysis of early auxin-responsive *Aux*/*IAA* gene family in rice (*Oryza sativa*). Funct Integr Genomics 6: 47–59. 16200395 10.1007/s10142-005-0005-0

[8] Hagen G, Guilfoyle T (2002) Auxin-responsive gene expression: genes, promoters and regulatory factors. Plant Mol Biol 49: 373–385. 12036261

[9] Arase F, Nishitani H, Egusa M, Nishimoto N, Sakurai S, et al (2012) IAA8 Involved in Lateral Root Formation Interacts with the TIR1 Auxin Receptor and ARF Transcription Factors in *Arabidopsis* . PLoS ONE 7: e43414. 22912871 10.1371/journal.pone.0043414 PMC3422273

[10] Tiwari SB, Hagen G, Guilfoyle TJ (2004) Aux/IAA Proteins Contain a Potent Transcriptional Repression Domain. Plant Cell 16: 533–543. 14742873 10.1105/tpc.017384 PMC341922

[11] Kagale S, Rozwadowski K (2011) EAR motif-mediated transcriptional repression in plants: An underlying mechanism for epigenetic regulation of gene expression. Epigenetics 6: 141–146. 20935498 10.4161/epi.6.2.13627 PMC3278782

[12] Szemenyei H, Hannon M, Long JA (2008) TOPLESS Mediates Auxin-Dependent Transcriptional Repression During Arabidopsis Embryogenesis. Science 319: 1384–1386. 18258861 10.1126/science.1151461

[13] Guilfoyle TJ, Hagen G (2012) Getting a grasp on domain III/IV responsible for Auxin Response Factor–IAA protein interactions. Plant Sci 190: 82–88. 22608522 10.1016/j.plantsci.2012.04.003

[14] Korasick DA, Westfall CS, Lee SG, Nanao MH, Dumas R, et al.. (2014) Molecular basis for AUXIN RESPONSE FACTOR protein interaction and the control of auxin response repression. Proc Natl Acad Sci USA: Early edition. 10.1073/pnas.1400074111 PMC3986151 24706860

[15] Rouse D, Mackay P, Stirnberg P, Estelle M, Leyser O (1998) Changes in Auxin Response from Mutations in an *AUX/IAA* Gene. Science 279: 1371–1373. 9478901 10.1126/science.279.5355.1371

[16] Tian Q, Reed JW (1999) Control of auxin-regulated root development by the Arabidopsis thaliana *SHY2/IAA3* gene. Development 126: 711–721. 9895319 10.1242/dev.126.4.711

[17] Rogg LE, Lasswell J, Bartel B (2001) A Gain-of-Function Mutation in *IAA28* Suppresses Lateral Root Development. Plant Cell 13: 465–480. 11251090 10.1105/tpc.13.3.465 PMC135515

[18] von Behrens I, Komatsu M, Zhang Y, Berendzen KW, Niu X, et al (2011) *Rootless with undetectable meristem 1* encodes a monocot-specific AUX/IAA protein that controls embryonic seminal and post-embryonic lateral root initiation in maize. Plant J 66: 341–353. 21219511 10.1111/j.1365-313X.2011.04495.x

[19] Worley CK, Zenser N, Ramos J, Rouse D, Leyser O, et al (2000) Degradation of Aux/IAA proteins is essential for normal auxin signalling. Plant J 21: 553–562. 10758506 10.1046/j.1365-313x.2000.00703.x

[20] Overvoorde P, Fukaki H, Beeckman T (2010) Auxin Control of Root Development. Cold Spring Harbor Perspectives in Biology. 10.1101/cshperspect.a001537 PMC2869515 20516130

[21] Rinaldi M, Liu J, Enders T, Bartel B, Strader L (2012) A gain-of-function mutation in IAA16 confers reduced responses to auxin and abscisic acid and impedes plant growth and fertility. Plant Mol Biol 79: 359–373. 22580954 10.1007/s11103-012-9917-y PMC3382072

[22] Tatematsu K, Kumagai S, Muto H, Sato A, Watahiki MK, et al (2004) *MASSUGU2* Encodes Aux/IAA19, an Auxin-Regulated Protein That Functions Together with the Transcriptional Activator NPH4/ARF7 to Regulate Differential Growth Responses of Hypocotyl and Formation of Lateral Roots in *Arabidopsis thaliana* . Plant Cell 16: 379–393. 14729917 10.1105/tpc.018630 PMC341911

[23] Uehara T, Okushima Y, Mimura T, Tasaka M, Fukaki H (2008) Domain II Mutations in CRANE/IAA18 Suppress Lateral Root Formation and Affect Shoot Development in *Arabidopsis thaliana* . Plant Cell Physiol 49: 1025–1038. 18505759 10.1093/pcp/pcn079

[24] Yang X, Lee S, So J-h, Dharmasiri S, Dharmasiri N, et al (2004) The IAA1 protein is encoded by AXR5 and is a substrate of SCFTIR1. Plant J 40: 772–782. 15546359 10.1111/j.1365-313X.2004.02254.x

[25] Fukaki H, Tameda S, Masuda H, Tasaka M (2002) Lateral root formation is blocked by a gain-of-function mutation in the *SOLITARY-ROOT/IAA14* gene of *Arabidopsis* . Plant J 29: 153–168. 11862947 10.1046/j.0960-7412.2001.01201.x

[26] Hamann T, Mayer U, Jurgens G (1999) The auxin-insensitive *bodenlos* mutation affects primary root formation and apical-basal patterning in the Arabidopsis embryo. Development 126: 1387–1395. 10068632 10.1242/dev.126.7.1387

[27] Nagpal P, Walker LM, Young JC, Sonawala A, Timpte C, et al (2000) *AXR2* Encodes a Member of the Aux/IAA Protein Family. Plant Physiol 123: 563–574. 10859186 10.1104/pp.123.2.563 PMC59024

[28] Ouellet F, Overvoorde PJ, Theologis A (2001) IAA17/AXR3: Biochemical Insight into an Auxin Mutant Phenotype. Plant Cell 13: 829–841. 11283339 10.1105/tpc.13.4.829 PMC135541

[29] Nakamura A, Umemura I, Gomi K, Hasegawa Y, Kitano H, et al (2006) Production and characterization of auxin-insensitive rice by overexpression of a mutagenized rice IAA protein. Plant J 46: 297–306. 16623891 10.1111/j.1365-313X.2006.02693.x

[30] Kitomi Y, Inahashi H, Takehisa H, Sato Y, Inukai Y (2012) OsIAA13-mediated auxin signaling is involved in lateral root initiation in rice. Plant Sci 190: 116–122. 22608525 10.1016/j.plantsci.2012.04.005

[31] Schnable JC, Springer NM, Freeling M (2011) Differentiation of the maize subgenomes by genome dominance and both ancient and ongoing gene loss. Proc Natl Acad Sci USA 108: 4069–4074. 21368132 10.1073/pnas.1101368108 PMC3053962

[32] Sheen J (2002) A transient expression assay using maize mesophyll protoplasts. http://geneticsmghharvardedu/sheenweb/.

[33] Nguyen HP, Chakravarthy S, Velasquez AC, McLane HL, Zeng LR, et al (2010) Methods to Study PAMP-Triggered Immunity Using Tomato and *Nicotiana benthamiana* . Mol Plant-Microbe Interact 23: 991–999. 20615110 10.1094/MPMI-23-8-0991

[34] Walter M, Chaban C, Schütze K, Batistic O, Weckermann K, et al (2004) Visualization of protein interactions in living plant cells using bimolecular fluorescence complementation. Plant J 40: 428–438. 15469500 10.1111/j.1365-313X.2004.02219.x

[35] Li M, Doll J, Weckermann K, Oecking C, Berendzen KW, et al (2010) Detection of in vivo interactions between Arabidopsis class A-HSFs, using a novel BiFC fragment, and identification of novel class B-HSF interacting proteins. Eur J Cell Biol 89: 126–132. 19945192 10.1016/j.ejcb.2009.10.012

[36] Berendzen K, Bohmer M, Wallmeroth N, Peter S, Vesic M, et al (2012) Screening for in planta protein-protein interactions combining bimolecular fluorescence complementation with flow cytometry. Plant Meth 8: 25. 10.1186/1746-4811-8-25 PMC3458939 22789293

[37] Edwards D, Berry JJ (1987) The Efficiency of Simulation-Based Multiple Comparisons. Biometrics 43: 913–928. 3427176

[38] Gray WM, Kepinski S, Rouse D, Leyser O, Estelle M (2001) Auxin regulates SCFTIR1-dependent degradation of AUX/IAA proteins. Nature 414: 271–276. 11713520 10.1038/35104500

[39] Reed JW (2001) Roles and activities of Aux/IAA proteins in *Arabidopsis* . Trends Plant Sci 6: 420–425. 11544131 10.1016/s1360-1385(01)02042-8

[40] Abel S, Theologis A (1995) A polymorphic bipartite motif signals nuclear targeting of early auxin-inducible proteins related to PS-IAA4 from pea (*Pisum sativum*). Plant J 8: 87–96. 7655509 10.1046/j.1365-313x.1995.08010087.x

[41] Dharmasiri N, Dharmasiri S, Estelle M (2005) The F-box protein TIR1 is an auxin receptor. Nature 435: 441–445. 15917797 10.1038/nature03543

[42] Abel S, Oeller PW, Theologis A (1994) Early auxin-induced genes encode short-lived nuclear proteins. Proc Natl Acad Sci USA 91: 326–330. 8278386 10.1073/pnas.91.1.326 PMC42940

[43] Ramos JA, Zenser N, Leyser O, Callis J (2001) Rapid degradation of auxin/indoleacetic acid proteins requires conserved amino acids of domain II and is proteasome dependent. Plant Cell 13: 2349–2360. 11595806 10.1105/tpc.010244 PMC139163

[44] Dreher KA, Brown J, Saw RE, Callis J (2006) The Arabidopsis Aux/IAA Protein Family has Diversified in Degradation and Auxin Responsiveness. Plant Cell 18: 699–714. 16489122 10.1105/tpc.105.039172 PMC1383644

[45] Padmanabhan MS, Goregaoker SP, Golem S, Shiferaw H, Culver JN (2005) Interaction of the Tobacco Mosaic Virus Replicase Protein with the Aux/IAA Protein PAP1/IAA26 Is Associated with Disease Development. J Virol 79: 2549–2558. 15681455 10.1128/JVI.79.4.2549-2558.2005 PMC546588

[46] Muto H, Watahiki MK, Nakamoto D, Kinjo M, Yamamoto KT (2007) Specificity and Similarity of Functions of the Aux/IAA Genes in Auxin Signaling of Arabidopsis Revealed by Promoter-Exchange Experiments among MSG2/IAA19, AXR2/IAA7, and SLR/IAA14. Plant Physiol 144: 187–196. 17369427 10.1104/pp.107.096628 PMC1913803

[47] Hamann T, Benkova E, Bäurle I, Kientz M, Jürgens G (2002) The Arabidopsis *BODENLOS* gene encodes an auxin response protein inhibiting MONOPTEROS-mediated embryo patterning. Genes Dev 16: 1610–1615. 12101120 10.1101/gad.229402 PMC186366

[48] Tao L-z, Cheung AY, Nibau C, Wu H-m (2005) RAC GTPases in Tobacco and Arabidopsis Mediate Auxin-Induced Formation of Proteolytically Active Nuclear Protein Bodies That Contain AUX/IAA Proteins. Plant Cell 17: 2369–2383. 15994909 10.1105/tpc.105.032987 PMC1182495

[49] Coll NS, Epple P, Dangl JL (2011) Programmed cell death in the plant immune system. Cell Death Differ 18: 1247–1256. 21475301 10.1038/cdd.2011.37 PMC3172094

[50] Kaminaka H, Näke C, Epple P, Dittgen J, Schütze K, et al (2006) bZIP10-LSD1 antagonism modulates basal defense and cell death in *Arabidopsis* following infection. EMBO J 25: 4400–4411. 16957775 10.1038/sj.emboj.7601312 PMC1570446

[51] Tiwari SB, Wang X-J, Hagen G, Guilfoyle TJ (2001) AUX/IAA Proteins Are Active Repressors, and Their Stability and Activity Are Modulated by Auxin. Plant Cell 13: 2809–2822. 11752389 10.1105/tpc.010289 PMC139490

[52] Kim J, Harter K, Theologis A (1997) Protein–protein interactions among the Aux/IAA proteins. Proc Natl Acad Sci USA 94: 11786–11791. 9342315 10.1073/pnas.94.22.11786 PMC23574

