# Supplementary figures and images for: Diversity of Stability, Localization, Interaction and Control of Downstream Gene Activity in the Maize Aux/IAA Protein Family
Source: PLoS One. 2014 Sep 9;9(9):e107346. doi: 10.1371/journal.pone.0107346 (PMC4159291; doi:10.1371/journal.pone.0107346)

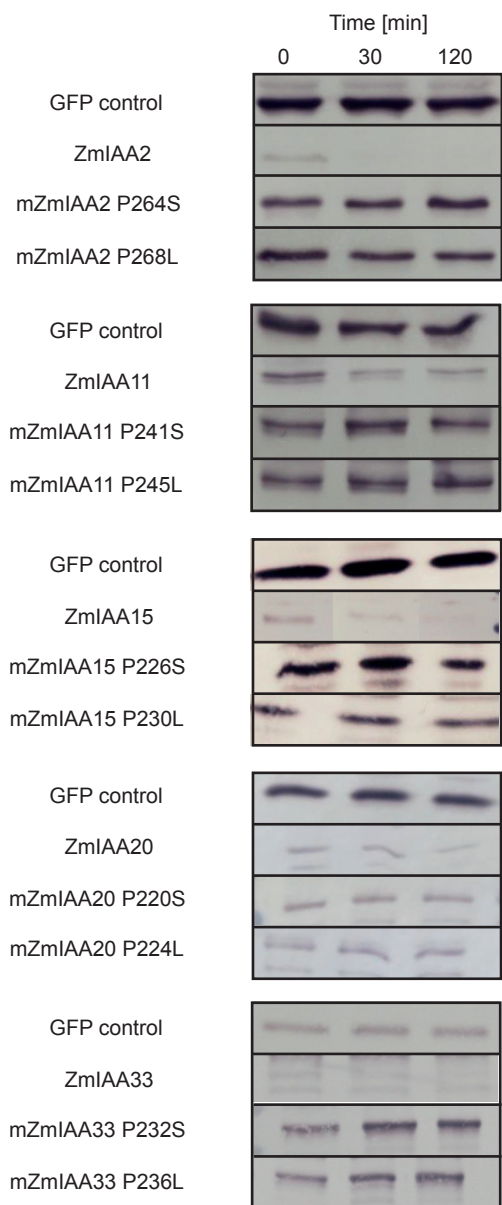

Supplement: Figure S2 — Confirmation of Aux/IAA degradation by Western blot experiments. Western blot analysis of Aux/IAA-GFP constructs after 1-NAA (10 µM) and cycloheximide (100 µg/ml) treatment using an anti-HA antibody. Protein abundance was quantified at three different time points (0, 30 and 120 min). Lane order for ZmIAA15 was manually rearranged to correct for the initial omission of the 30 min sample which was loaded on the same gel in a lane to the right. (PDF) [file pone.0107346.s002.pdf]

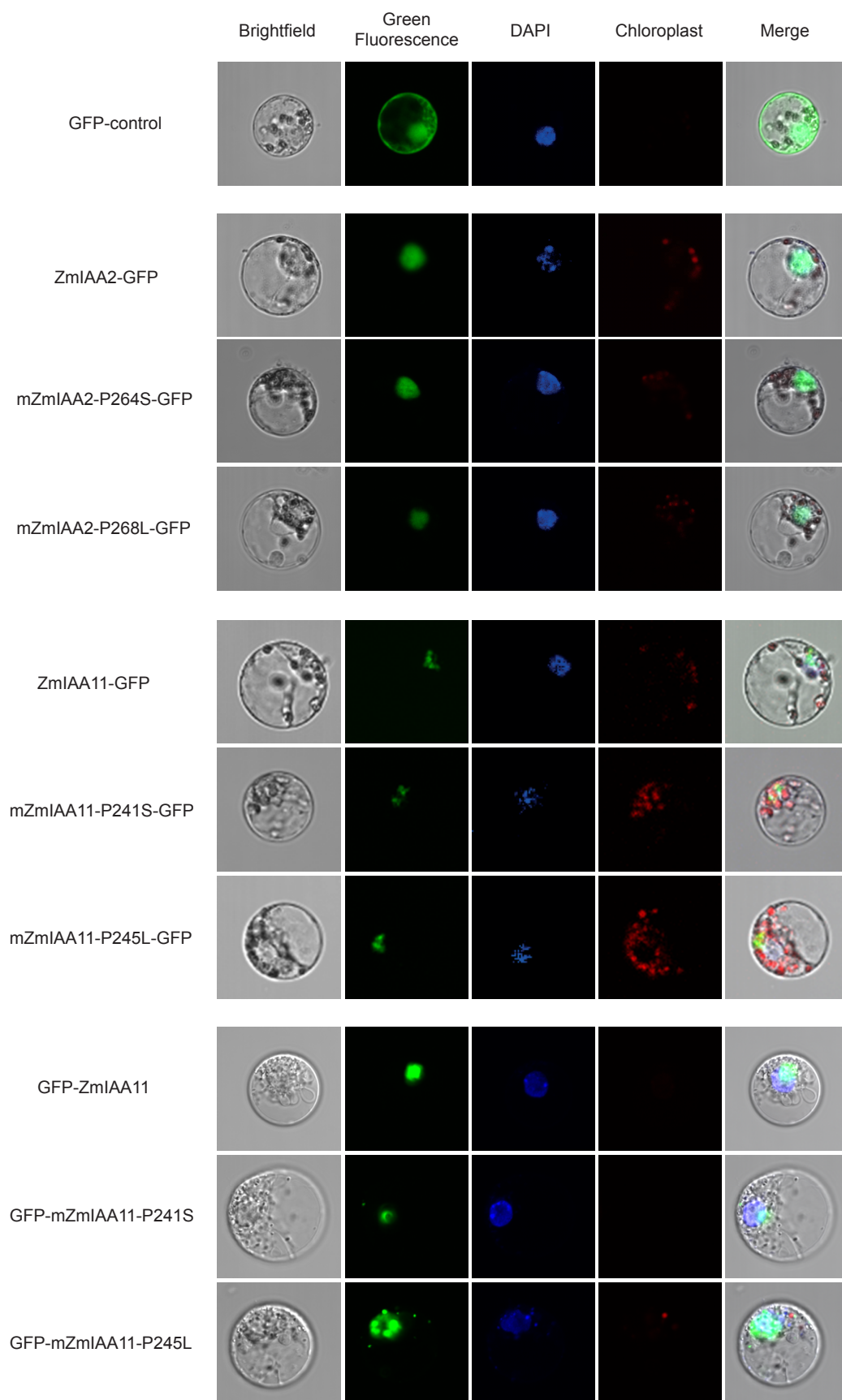

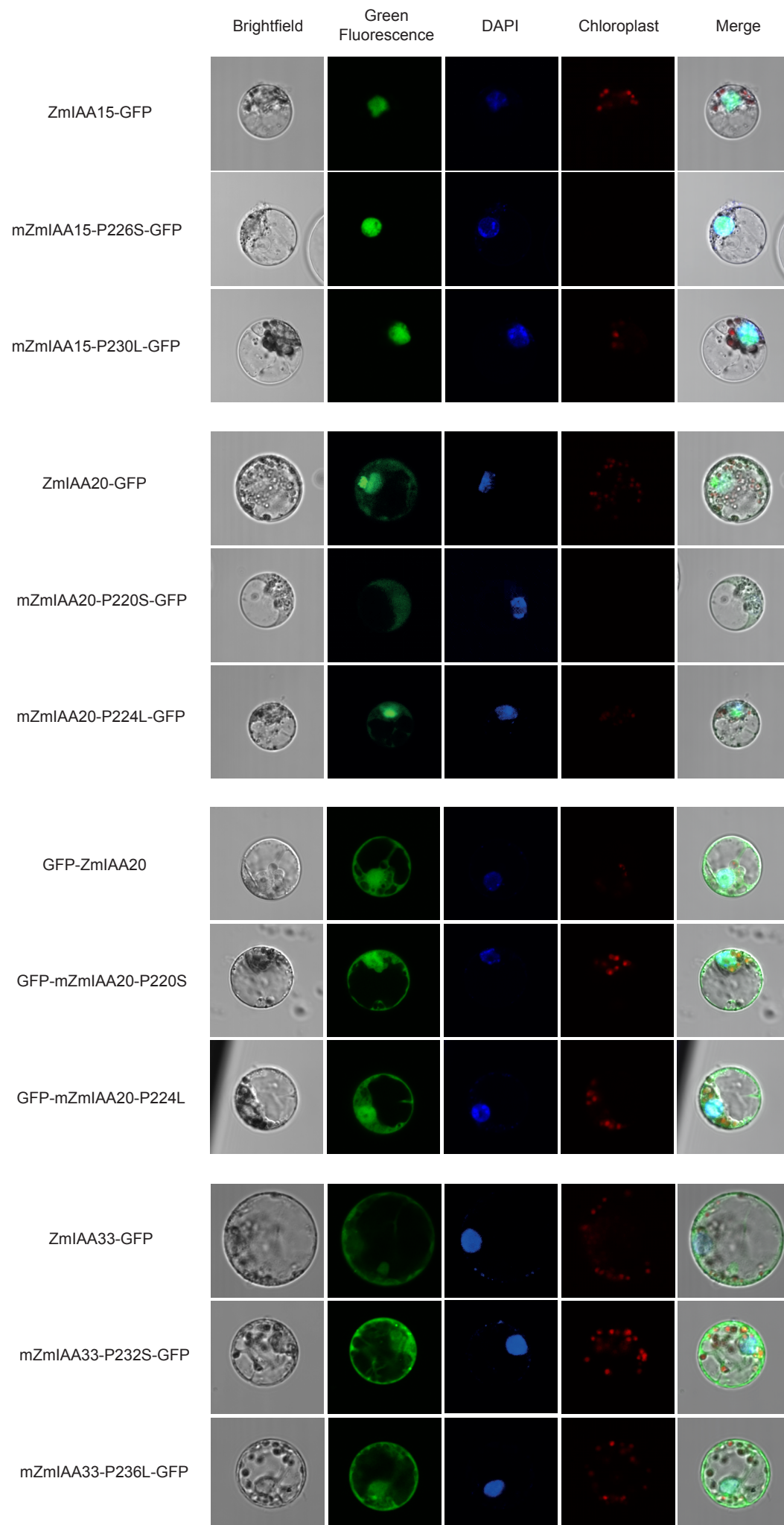

Supplement: Figure S3 — Subcellular localization studies of maize Aux/IAA proteins and their mutated forms in maize protoplasts. Detailed summary of the subcellular localization studies of maize Aux/IAA wild-type proteins and two mutated protein forms of each protein in maize protoplasts. As a control, the empty GFP construct constitutively expressing GFP, was localized in the cytosol and nucleus. ZmIAA2, ZmIAA11 and ZmIAA15 were confined to the nucleus. The specific point-mutations in the degron-sequence did not affect localization. For ZmIAA20 and ZmIAA33, wild-type and mutated proteins were localized in the cytosol and the nucleus. To investigate if the compartmentalization of the GFP signal is a result of C-terminal GFP, N-terminal GFP fusion constructs of ZmIAA11 and ZmIAA20 with their mutated forms were tested. The localization study displayed an accumulation in a compartmental manner in the nucleus as described before. (PDF) [file pone.0107346.s003.pdf]

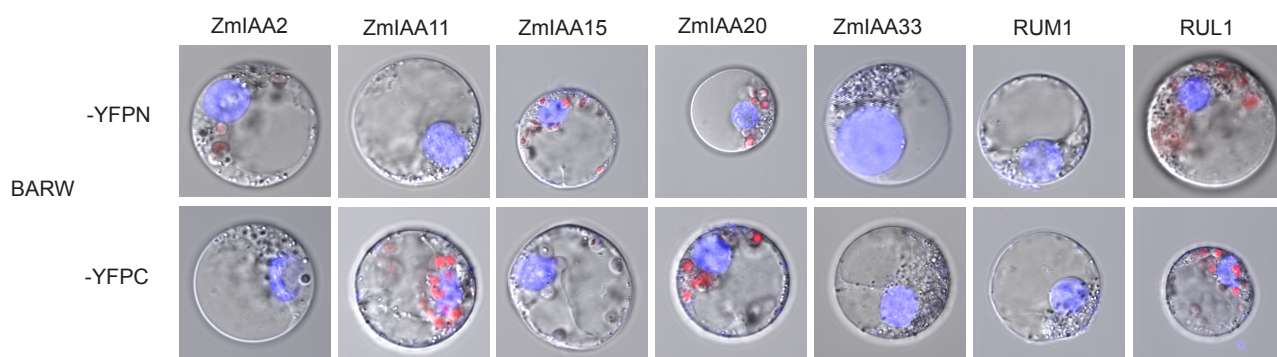

Supplement: Figure S4 — Negative controls for protein-protein interaction studies in maize protoplasts. In maize protoplasts split-YFP experiments were conducted to demonstrate that Aux/IAA proteins do not interact with the control protein BARW. Red: auto fluorescence, blue: DAPI counterstaining. (PDF) [file pone.0107346.s004.pdf]

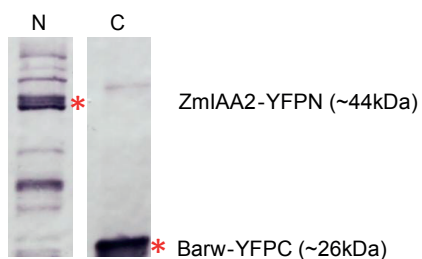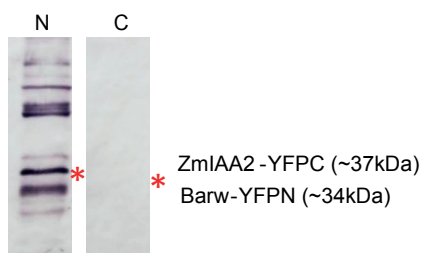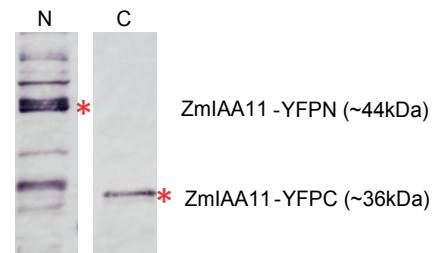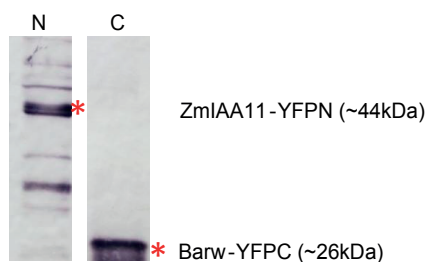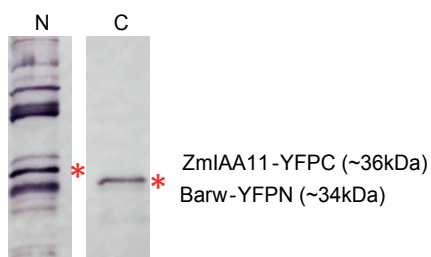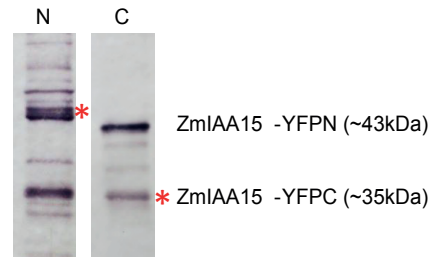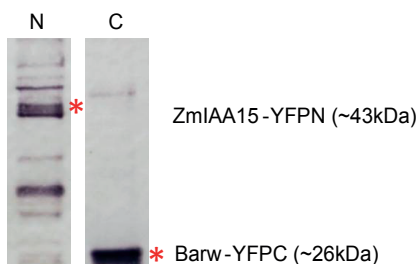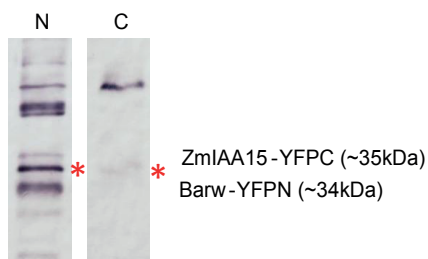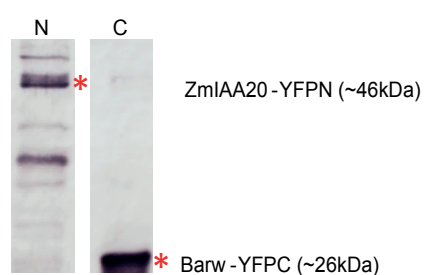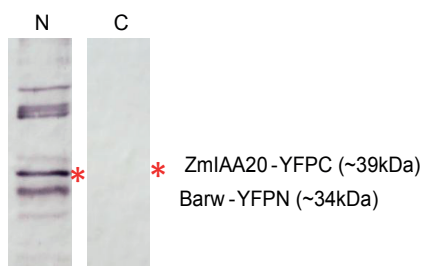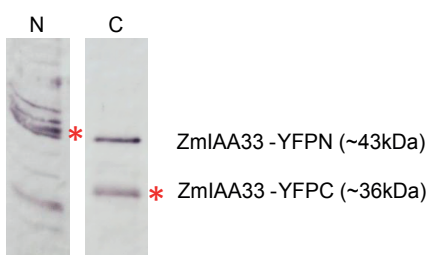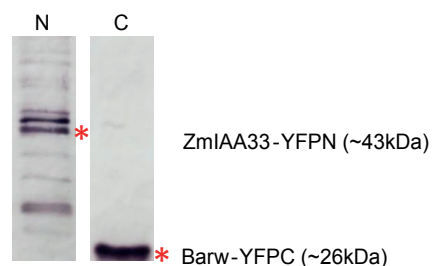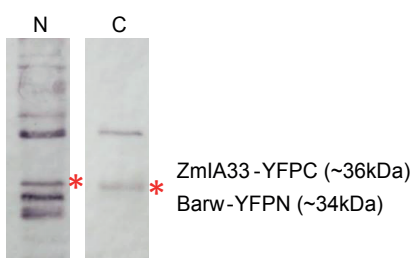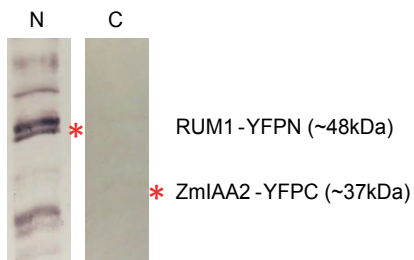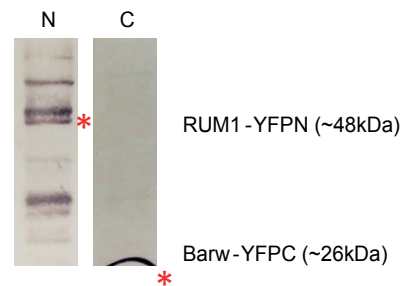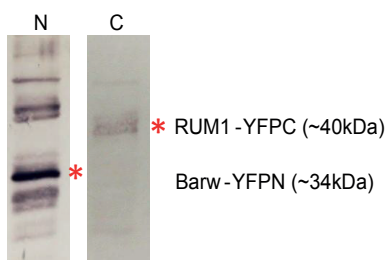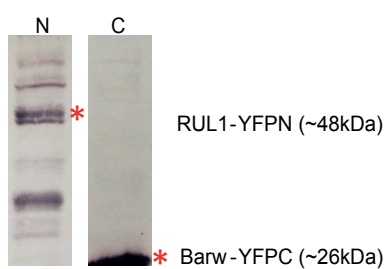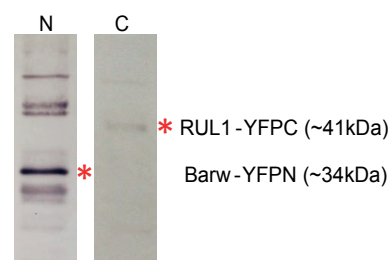

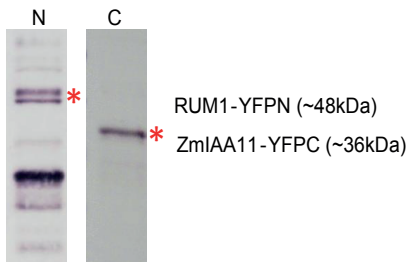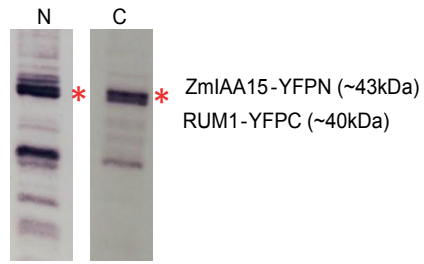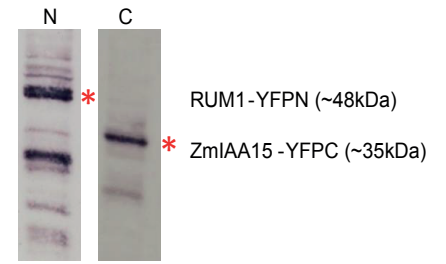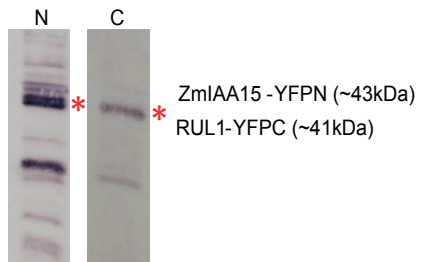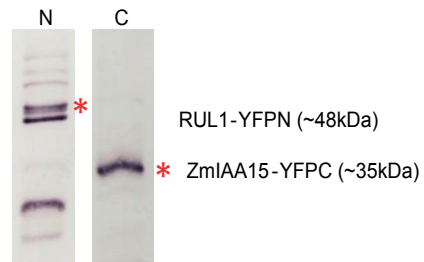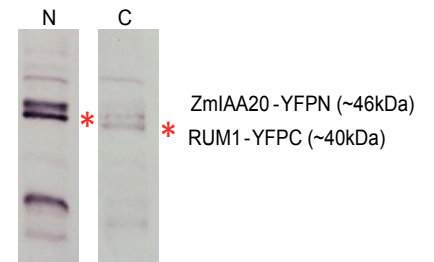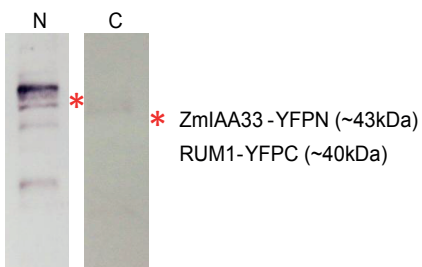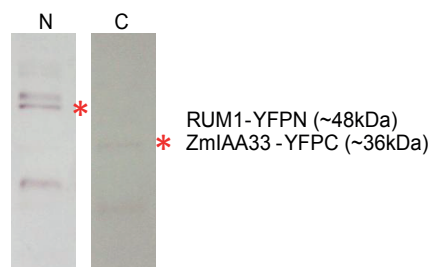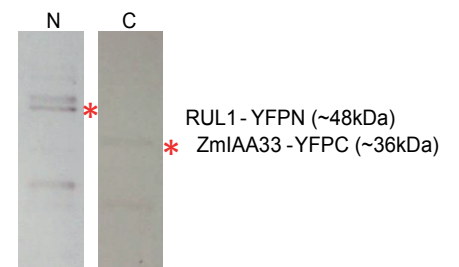

Supplement: Figure S5 — Confirmation of fusion protein expression in protein-protein interaction studies. Expression of the fusion proteins was demonstrated by Western blot experiments. The positions of the expressed proteins are indicated with red stars to the right of the corresponding lane. Anti-Myc antibodes were used for the detection of YFPN-152 and anti-HA for the detection of YFPC. (PDF) [file pone.0107346.s005.pdf]
